# Paternal Health and Health Behaviors During the Perinatal Period: Results from a Representative Survey of Fathers in Georgia, 2018–2019

**DOI:** 10.1007/s10995-025-04090-x

**Published:** 2025-04-29

**Authors:** Raj M. Dalal, Clarissa D. Simon, John James Parker, Anne Bendelow, Michael Bryan, Craig F. Garfield

**Affiliations:** 1 Department of Pediatrics, McGaw Medical Center Northwestern University Feinberg School of Medicine, 420 E. Superior, Chicago, IL 60611 USA; 2 Family and Child Health Innovations Program, Research and Evaluation Center, Smith Child Health Outcomes, Ann & Robert H. Lurie Children’s Hospital of Chicago, Chicago, IL USA; 3 Data Analytics and Reporting, Ann & Robert H. Lurie Children’s Hospital of Chicago, Chicago, IL USA; 4 Department of Maternal and Child Health Epidemiology, Georgia Department of Public Health, Atlanta, GA USA

**Keywords:** Fathers, Healthcare utilization, Paternal health, Family health, Public health

## Abstract

**Objectives:**

To investigate the associations between paternal sociodemographic characteristics, healthcare utilization and self-reported health status among a state-representative sample of fathers.

**Methods:**

The Pregnancy Risk Assessment Monitoring System for Dads pilot study sampled 857 fathers in Georgia from October 2018-July 2019. It surveyed fathers 2–6 months after their infants’ birth to assess paternal experiences and behaviors during the perinatal period. Multivariable logistic regression examined associations between paternal characteristics and three outcomes: having a primary care physician (PCP), having any personal healthcare visit, and self-reported health status.

**Results:**

Among 266 respondents, 53.9% reported having a PCP, 46.2% reported any healthcare visit, and 65.2% reported very good or excellent health. Insured fathers were more likely to have a PCP (65.6% vs. 26.6%; adjusted Prevalence Ratio [aPR] = 2.47, 95% CI 1.41–4.33) and a healthcare visit (59.9% vs. 21.5%; aPR = 2.60, 95% CI 1.30–5.22) than fathers who were uninsured. Fathers with a college degree or higher were more likely to have a healthcare visit (59.4% vs. % 39.3%; aPR = 1.68, 95% CI 1.13–2.49), and to report very good or excellent health (79.1% vs. % 52.2%; aPR = 1.52, 95% CI 1.16–1.98) than fathers with a high school diploma/GED or less. Fathers reporting very good or excellent health were more likely to have a PCP (59.9% vs. 42.1%); aPR = 1.42, 95% CI 1.02–1.99) than fathers reporting fair or good health.

**Conclusions:**

Fathers’ participation in healthcare was suboptimal. Identifying barriers impacting men’s interactions with the healthcare system is essential to develop strategies to improve the overall health of fathers and families.

## Significance

What is Already Known on this Subject?

What this Study Adds?

Men have lower healthcare engagement and utilization compared with women, which may lead to adverse health outcomes for men who are fathers, their partners, and their infants.

Our findings highlight the interrelationship between fathers’ health and access to and use of health and healthcare services during the perinatal period. Examining the impact of these health measures on fathers may lead to innovative approaches to improve paternal and family health.

## Objectives

Prior research on the effects of fatherhood on men’s health suggests that entrance into fatherhood is associated with changes in physical and mental health (Garfield et al., 2014, 2016; Umberson et al., 2017; Yogman et al., 2016). As a key life course event, the perinatal period has been documented as a potential window to improve paternal health, behaviors, and psychological well-being (Garfield et al., 2010). In addition, it is an opportunity to address health disparities for men’s health, and by extension potentially improve maternal and child health (Boykin et al., 2020; Jack et al., 2008; Salvesen von Essen et al., 2021; Yogman et al., 2016). Markers of poor paternal health during the perinatal period are linked to poor child health and maternal health (Azuine & Singh, 2019; Cui et al., 2020; Freeman et al., 2012; Paulson & Bazemore, 2010), including associations between paternal wellbeing and child wellbeing (Scarlett et al., 2024; Yogman et al., 2016), yet there are limited data on objective and subjective metrics for paternal health status and healthcare utilization.

Despite the critical role that fathers play in the health and development of their families, male health in the United States continues to lag behind their female counterparts. Recent data from the Centers for Disease Control and Prevention’s National Center for Health Statistics (NCHS) reveals that men have a higher all-cause mortality rate and lower life expectancy than women (National Center for Health Statistics, 2023). Lower healthcare utilization is one known contribution to these health disparities as fewer visits to physicians were made by males than females, a difference that was most pronounced among adults aged 18–44 with males (National Center for Health Statistics, 2023). Improving healthcare utilization, especially primary care, is known to improve health outcomes and mitigate health disparities as it allows for prevention, early identification, and longitudinal management of diseases with significant morbidity and mortality risk such as diabetes and hypertension (Azuine & Singh, 2019). This is recognized by the Healthy People 2030 initiative, which outlines the goal of increasing the proportion of people with a usual primary care provider from 76% in 2017 to 84% by 2030.

Poor healthcare utilization and healthcare behaviors by fathers during the perinatal period not only adversely impacts the health of the father, it can have downstream effects on his partner, and his offspring, particularly with respect to mental health (Azuine & Singh, 2019; Cui et al., 2020; Paulson & Bazemore, 2010). A focus on the importance of connecting fathers with the healthcare system and preventive healthcare may therefore provide a three-way benefit to fathers, mothers, and infants. To drive research, community, and clinical initiatives aimed at improving health and healthcare utilization for fathers, data is needed to better inform these interventions and approaches.

Although population-based maternal public health surveillance systems have been in place for decades (Kortsmit et al., 2021), no large-scale national public health surveillance system currently exists dedicated to paternal health and health behaviors in the perinatal period (Garfield et al., 2022). This pilot study examines the overall prevalence and associations of select paternal characteristics and three paternal healthcare outcomes during the perinatal period: (1) having a primary care physician (PCP), (2) having any type of personal healthcare visit during the perinatal period, and (3) self-reported health status.

## Methods

### Data Source

Data was analyzed from the Pregnancy Risk Assessment Monitoring System (PRAMS) for Dads, a novel population-based cross-sectional study piloted in the state of Georgia (GA) in 2018–2019 (Garfield et al., 2022). The methodology of PRAMS for Dads has been further described elsewhere, with PRAMS for Dads contact protocols aligned with PRAMS (Garfield et al., 2022). Fathers were sampled if the infant’s mother had been randomly sampled from a list of birth certificates for recent live births (Kortsmit et al., 2021). For this pilot, fathers were sampled during the period from October 15, 2018-July 3, 2019 (representing infants born during May 28, 2018-May 3, 2019). Fathers were surveyed 2 to 6 months following the birth of their infant (*n* = 857). Data on fathers was obtained from the infants’ birth certificates such as age, education, race and Hispanic origin, and marital status -- fathers were either married as identified on the birth certificate, or unmarried with a completed paternity acknowledgment form (PA). This study was conducted in accord with the ethical standards laid down in the 1964 Declaration of Helsinki and its later amendments. The study was approved by the Institutional Review Boards at Northwestern University and Georgia Department of Public Health (IRB of record).

### Paternal Characteristics

Paternal characteristics obtained from infant birth certificate records included: age (< 25, 25–34 or ≥ 35 years), education (≤ high school diploma or GED; some college, no degree; and college degree (associate degree, bachelor’s degree or higher), marital status (married or unmarried with PA), and race and Hispanic origin (non-Hispanic white, non-Hispanic Black, Hispanic, and Non-Hispanic other). Because of small sample size (*n* = 22), the category for “non-Hispanic, other” included all other reported races (e.g. Asian-Pacific Islander, multiple races, other) and unknown race and Hispanic origin. Information on health insurance status after the infants’ birth was obtained from responses to the question “What kind of health insurance do you have now?” where respondents could select all qualifying responses. Response options were categorized as either insured, if reporting having any type of health insurance (e.g. “Private health insurance from my job or the job of my wife or partner,” “Private health insurance from my parents,” “Private health insurance from the Health Insurance Marketplace or Healthcare.gov,” “Medicaid,” “PeachCare for Kids,” “TRICARE or other military health care,” and “Other health insurance”), or uninsured if they selected “I don’t have health insurance now”.

### Healthcare and Health Outcomes

Fathers having a healthcare visit during the perinatal period was assessed by answering affirmatively to “Did you have any healthcare visits with a doctor, nurse or other healthcare worker, including a dental or mental health worker?” in reference to either the time period “during your baby’s mother’s pregnancy” or “since your new baby was born.” Having a PCP was assessed by affirmative answers to either “Did you have a primary care physician when your baby’s mother was pregnant?” or “Do you have a primary care physician now?” Responses for these two time points were combined to create variables for any healthcare visit and any primary care physician during the perinatal period. Self-reported health status was based on response to the question, “Would you say that in general your health is:” with five answer options ranging from “Excellent” to “Poor.” This variable was then dichotomized as very good or excellent compared with good, fair, or poor to create two approximately equal groupings of fathers.

### Statistical Analyses

All analyses were conducted using STATA version 17 (StataCorp, College Station, Texas 77845 USA). We include descriptive statistics for paternal characteristics chosen due to associations with healthcare utilization for men (Schlichthorst et al., 2016; Vaidya et al., 2012) (e.g. age, race and Hispanic origin, health insurance status, education, and marital status), three main outcomes during the perinatal period (e.g. having a primary care physician, having a recent health care visit, and self-reported health status) and 95% confidence intervals. Multivariable logistic regression was used to measure associations between the previously described paternal characteristics and the three main outcomes. Healthcare utilization outcomes were examined (i.e., having a PCP and a recent healthcare visit) using self-reported health status as an independent variable to evaluate whether self-reported health status was associated with healthcare utilization outcomes. Adjusted prevalence ratios (aPR) were calculated using STATA’s post-estimation command adjrr (Norton et al., 2013). All analyses accounted for PRAMS for Dads complex survey sampling design and survey weights (sampling, nonresponse, and noncoverage weights) to be representative of fathers to live-born infants who were either married or unmarried, with a completed PA form in Georgia.

## Results

Among 266 respondents, 13.1% were less than 25 years old, 56.7% were 25–34 years old, and 30.2% were 35 years or older. Regarding race and Hispanic origin, 44.8% were non-Hispanic white, 28.2% were non-Hispanic Black, 18.9% were Hispanic, 8.2% reported being “non-Hispanic other.” In this sample, 69.6% were insured, 35.7% were college graduates, and 64.7% were married (Table 1). During the perinatal period, 53.9% having a PCP, 46.2% had any healthcare visit, and 65.2% reported their health was very good or excellent at survey completion. No respondents reported poor health.

**Table 1:** Prevalence of paternal characteristics and healthcare outcomes during the perinatal period, pregnancy risk assessment monitoring system for dads, 2018–2019

Characteristic	Unweighted sample size (*n* = 266)^a^	Weighted % (95% CI)^b^
**Age**		
< 25	31	13.1 (8.6–19.4)
25–34	144	56.7 (49.0-64.1)
≥ 35	91	30.2 (23.7–37.6)
**Race and Hispanic origin**		
Non-Hispanic white	121	44.8 (37.3–52.4)
Non-Hispanic Black	73	28.2 (21.6–35.8)
Hispanic	50	18.9 (13.7–25.6)
Non-Hispanic other or unidentified^c^	22	8.2 (4.8–13.6)
**Insurance Status**		
Uninsured	80	30.4 (23.7–38.2)
Insured	186	69.6 (61.8–76.3)
**Education**		
≤High school or GED	123	44.8 (37.4–52.5)
Some college, no degree	47	19.5 (13.8–26.8)
College graduate^d^	96	35.7 (28.8–43.2)
**Marital status**		
Unmarried with PA form	72	35.3 (28.0-43.5)
Married	193	64.7 (56.5–72.0)
**Have a primary care physician**		
No	131	46.1 (38.6–53.8)
Yes	134	53.9 (46.2–61.4)
**Any healthcare visit** ^**e**^		
No healthcare visit	141	53.8 (46.0-61.3)
Healthcare visit	123	46.2 (38.7–54.0)
**Self-reported health status (SRHS)**		
Good, fair, or poor health^f^	92	34.8 (27.7–42.6)
Very good or excellent	168	65.2 (57.4–72.3)

*Abbreviations* PA = Paternity Acknowledgement, GED = General Educational Development

^a^ Unweighted total sample size for variable; sample size may vary due to missing

^b^ Weighted percentage. Father analysis weight is the product of his sampling weight, non-response weight and non-coverage weight

^c^ Non-Hispanic other included all other reported races and fathers who left race unanswered

^d^ Associate degree, bachelor’s degree or higher

^e^For time period of “when baby’s mother was pregnant” or “since my new baby was born”

^f^No poor health reported

The prevalence of having a PCP (Table 2) during the perinatal period was higher among fathers who were insured compared with fathers who were uninsured (66.3% vs. 24.9%) and among fathers with a college degree compared with fathers with high school diploma/GED or less education (66.9% vs. 38.1%). Having at least one healthcare visit was also higher among fathers who were insured compared with fathers who were uninsured (59.3% vs. 16.0%) and among fathers with more education (66.7% vs. 30.3%). The prevalence of having at least one healthcare visit during the perinatal period was also higher among fathers who were non-Hispanic, white (55.5%) or non-Hispanic, Black (50.1%) compared with fathers who were Hispanic (18.1%).

**Table 2:** Prevalence of healthcare outcomes during the perinatal period by selected paternal characteristics, pregnancy risk assessment monitoring system for dads, 2018–2019 (*n* = 266)

Characteristic	Primary care physician		Any healthcare visit			Self-reported very good or excellent health	
	*n* ^a^	Weighted % (95% CI)^b^	*n* ^a^	Weighted % (95% CI)^b^		*n* ^a^	Weighted % (95% CI)^b^
**Overall**	265	53.9 (46.2–61.4)	264	46.2 (38.7–54.0)		260	65.2 (57.4–72.3)
**Age**							
< 25	31	38.9 (20.5–61.0)	31	36.9 (26.1–47.4)		30	62.0 (39.7–80.2)
25–34	143	52.9 (42.6–62.9)	143	43.5 (33.7–53.7)		142	63.4 (52.9–72.7)
≥ 35	91	62.2 (48.4–74.3)	90	55.6 (41.9–68.6)		88	70.1 (55.9–81.3)
**Race and Hispanic origin**							
Non-Hispanic white	121	62.8 (51.5–72.8)	121	55.5 (44.2–66.2)		121	64.5 (53.1–74.5)
Non-Hispanic Black	73	48.3 (33.7–63.2)	73	50.1 (35.4–64.8)		71	64.9 (48.6–78.3)
Hispanic	49	43.9 (27.8–61.4)	48	18.1 (8.5–34.6)		48	71.4 (53.8–84.3)
Non-Hispanic other or unidentified	22	46.8 (22.8–72.4)	22	43.6 (20.5–70.0)		20	56.3 (29.6–79.7)
**Insurance Status**							
Uninsured	79	24.9 (14.1–40.0)	79	16.0 (7.5–31.2)		80	57.7 (43.0-71.2)
Insured	186	66.3 (57.4–74.2)	185	59.3 (50.2–67.9)		180	68.6 (59.5–76.5)
**Education**							
≤High school diploma or GED	122	38.1 (27.8–49.7)	122	30.3 (20.8–41.8)		118	53.8 (42.1–65.1)
Some college, no degree	47	65.8 (47.0-80.6)	47	45.7 (28.4–64.1)		47	66.5 (47.4–81.4)
College graduate^c^	96	66.9 (54.4–77.5)	95	66.7 (53.8–77.4)		95	78.3 (66.2–87.0)
**Marital status**							
Unmarried with PA form	72	45.8 (31.8–60.7)	73	33.4 (21.0-48.6)		71	55.3 (40.4–69.3)
Married	193	58.2 (49.4–66.5)	191	53.4 (44.6–62.0)		189	70.7 (62.1–78.0)

*Abbreviations* PA = Paternity Acknowledgement, GED = General Educational Development

^a^ Unweighted total sample size for variable; sample size may vary due to missing

^b^ Weighted percentage. Father analysis weight is the product of his sampling weight, non-response weight and non-coverage weight

^c^ Associate degree, bachelor’s degree, or higher

The prevalence of reporting very good or excellent health was higher among fathers who were insured compared with fathers who were uninsured (68.6% vs. 57.7%), fathers with a college degree compared with a high school diploma/GED or less education (78.3% vs. 53.8%), and fathers who were married compared with unmarried with a PA (70.7% vs. 55.3%).

In multivariable regression models adjusted for age, race and Hispanic origin, insurance status, education, and marital status (Table 3), a higher prevalence of having a PCP during the perinatal period was reported among fathers who were insured compared with uninsured (65.6% vs. 26.6%; adjusted prevalence ratio [aPR]: 2.47, 95% CI: 1.41–4.33), and fathers with some college compared with fathers with a high school diploma/GED or less (69.0% vs. 42.3%; aPR: 1.63, 95% CI: 1.16–2.30). A higher prevalence of having at least one healthcare visit during the perinatal period was reported among fathers who were insured compared with uninsured (55.9% vs. 21.5%; aPR: 2.60, 95% CI: 1.30–5.22), and fathers with a college degree compared with a high school diploma/GED or less (59.4% vs. 45.1%; aPR: 1.68, 95% CI: 1.13–2.49) The prevalence of reporting very good or excellent health was higher among fathers with a college degree compared with fathers with a high school diploma/GED or less (79.1% vs. 52.2%; aPR = 1.52, 95% CI: 1.16–1.98).

**Table 3:** Adjusted prevalence and adjusted prevalence ratios for healthcare utilization and health status during the perinatal period by selected paternal demographic characteristics: pregnancy risk assessment monitoring system (PRAMS) for dads 2018–2019

Characteristic	Primary care physician		Any healthcare visit		Self-reported very good or excellent health	
	Adjusted Prevalence	aPR^b^ (95% CI)	Adjusted Prevalence	aPR^b^ (95% CI)	Adjusted Prevalence	aPR^b^ (95% CI)
**Age**						
< 25	41.0%	0.66 (0.39–1.11)	49.5%	0.98 (0.60–1.59)	67.0%	1.00 (0.69–1.44)
25–34	52.3%	0.84 (0.65–1.08)	43.3%	0.85 (0.62–1.17)	63.8%	0.95 (0.75–1.20)
≥ 35	62.5%	ref	50.7%	ref	67.3%	ref
**Race and Hispanic origin**						
Non-Hispanic white	56.7%	ref	48.6%	ref	60.6%	ref
Non-Hispanic Black	49.8%	0.88 (0.62–1.25)	56.6%	1.16 (0.82–1.65)	68.8%	1.14 (0.85–1.52)
Hispanic	60.1%	1.07 (0.80–1.42)	28.5%	0.59 (0.31–1.10)	77.4%	1.28 (0.99–1.65)
Non-Hispanic other or unidentified	38.2%	0.67 (0.38–1.20)	31.6%	0.65 (0.31–1.35)	45.4%	0.75 (0.41–1.36)
**Insurance Status**						
Uninsured	26.6%	ref	21.5%	ref	61.5%	ref
Insured	65.6%	2.47 (1.41–4.33)	55.9%	2.60 (1.30–5.22)	67.1%	1.09 (0.82–1.46)
**Education**						
≤High school diploma or GED	42.3%	ref	35.3%	ref	52.2%	ref
Some college, no degree	69.0%	1.63 (1.16–2.30)	45.1%	1.28 (0.77–2.10)	68.3%	1.31 (0.94–1.83)
College graduate^c^	59.6%	1.41 (0.98–2.03)	59.4%	1.68 (1.13–2.49)	79.1%	1.52 (1.16–1.98)
**Marital status**						
Unmarried with PA form	57.1%	0.91 (0.67–1.24)	43.5%	1.10 (0.75–1.61)	59.0%	1.17 (0.87–1.58)
Married	52.2%	ref	47.7%	ref	69.0%	ref

*Abbreviations* PA = Paternity Acknowledgement, GED = General Educational Development, aPR = adjusted Prevalence Ratio

^b^ All models adjusted for age, race and Hispanic origin, insurance status, education, and marital status

^c^ Associate degree, bachelor’s degree, or higher

**Table 4:** Adjusted prevalence and adjusted prevalence ratios for healthcare utilization by self-reported paternal health status during the perinatal period: pregnancy risk assessment monitoring system (PRAMS) for dads 2018–2019

	Primary care physician		Any healthcare visit	
	Adjusted prevalence	aPR^a^ (95% CI)	Adjusted prevalence	aPR^a^ (95% CI)
**Self-reported health status**				
Very good or excellent	59.9%	1.42 (1.02–1.99)	46.8%	1.06 (0.76–1.48)
Good or fair	42.1%	ref	44.2%	ref

*Abbreviations* aPR = adjusted Prevalence Ratio

^a^All models adjusted for age, race and Hispanic origin, insurance status, education and marital status

When examining the associations between healthcare utilization (recent healthcare visit, having a PCP), and self-reported health status we observed no differences in the prevalence of having a healthcare visit when comparing fathers who reported very good or excellent health compared with fathers reporting good or fair health (46.8% vs. 44.2%; aPR:1.06, 95% CI: 0.76–1.48). However, a higher prevalence of having a PCP was reported among fathers who reported their health was very good or excellent compared with fathers who reported their health was good or fair (59.9% vs. 42.1%, aPR:1.42, 95% CI: 1.02–1.99).

## Discussion

In this state-representative study of fathers in Georgia, we examined the associations between fathers’ healthcare utilization and health status by selected paternal characteristics during a key moment in fathers’ life course: the transition to fatherhood. Certain groups reported a lower prevalence of healthcare utilization and poorer self-reported health and may be a focus for future programs and interventions. These groups include fathers without health insurance who were significantly less likely to have a PCP or at least one recent healthcare visit than fathers with health insurance; likewise, fathers with a high school diploma/GED were less likely to have a PCP or at least one recent healthcare visit than fathers with a college degree, and more likely to have poorer self-reported health status. These findings could guide providers in creating community-based interventions to improve utilization of care for these at-risk subpopulations to promote more equitable and accessible care for the entire family. For example, improving opportunities for health insurance access for uninsured men during the perinatal period may benefit fathers by making health care more accessible and less costly (Salvesen von Essen et al., 2021).

Identifying key life course events for men as areas of focus for both community and clinically-based interventions is essential for public health. The perinatal period may offer unique opportunities to improve healthcare utilization for men transitioning into fatherhood (Salvesen von Essen et al., 2021). Our analysis further demonstrates the link between improved health and healthcare utilization as noted by the association between fathers reporting better health and having a PCP. Health behaviors of fathers during the perinatal period may extend throughout adulthood and can impact men’s health outcomes long term.

Prior studies highlight that the perinatal period can be an opportune time to intervene for the health benefit of new fathers. A qualitative study of urban fathers in the Midwest found that entrance into fatherhood coincided with a positive adjustment in health behaviors and attitudes out of a desire to live to see their child reach adulthood and to serve as a role model for their child (Garfield et al., 2010). Several innovative community-based initiatives capitalize on fatherhood as a lever for lifestyle change that begins with the father and continues to the larger family. One lifestyle health intervention “Healthy Dads, Healthy Kids’’ aims to assist fathers in their weight loss goals while influencing the lifestyle behaviors of their children through weekly educational group sessions taught by physical education teachers. Results of the randomized trial found that fathers of the program had significantly greater weight loss and both they and their children had greater physical activity levels (Morgan et al., 2014). Additionally, “Fathers and Babies,” a mental health intervention piloted in the family’s home through a home-visiting program, utilizes cognitive-behavioral therapy techniques for fathers in the perinatal period and reported significant decreases in fathers perceived stress 3 and 6 month follow up from baseline (Tandon et al., 2021). Engaging fathers in healthcare and optimal health behaviors, especially during the perinatal period, may help them improve their own health as well as their families.

Limitations exist for this study. The cross-sectional design limits the interpretation of any associations drawn between paternal characteristics and fathers’ healthcare utilization and self-reported health status. The survey design of this study may also increase the risk for response and recall bias as fathers completed the survey at one specific time point to report behaviors encompassing the pre- and postnatal period. Furthermore, the generalizability of these findings may be limited to fathers in Georgia. Finally, while the response rate (31.7%) was low, this is a pilot study and it is consistent with other surveys of fathers (Avenilla et al., 2006; Carlson & McLanahan, 2010). Despite these limitations, PRAMS for Dads is a novel population-based public health surveillance system that captures a snapshot of fathers’ utilization of healthcare and health during the perinatal period.

## Conclusions

Fathers in this state-representative sample generally reported low healthcare utilization during the perinatal period. In particular, select paternal characteristics such as health insurance and education appear linked to healthcare utilization, which could contribute to the health status of fathers and have downstream impacts on families. The perinatal period may be perceived as a lever for change and used as an opportunity for programming that focuses on the health of prospective and new fathers. As data has shown that expectant fathers tend to pay more attention to their health (Garfield et al., 2010), the perinatal period may serve as a key inflection point to increase public health messaging and interventions around men’s health.
